# Multi-Omics Analysis Reveals the Resistance Mechanism and the Pathogens Causing Root Rot of *Coptis chinensis*

**DOI:** 10.1128/spectrum.04803-22

**Published:** 2023-02-21

**Authors:** Xuhong Song, Pengying Mei, Tao Dou, Qundong Liu, Longyun Li

**Affiliations:** a Chongqing Academy of Chinese Materia Medica, Chongqing, People’s Republic of China; University of North Dakota

**Keywords:** root rot, *Coptis chinensis*, resistance, pathogen, multi-omics

## Abstract

*Coptis chinensis* is a traditional Chinese medicinal herb used for more than 2,000 years. Root rot in *C. chinensis* can cause brown discoloration (necrosis) in the fibrous roots and rhizomes, leading to plants wilting and dying. However, little information exists about the resistance mechanism and the potential pathogens of the root rot of *C. chinensis* plants. As a result, in order to investigate the relationship between the underlying molecular processes and the pathogenesis of root rot, transcriptome and microbiome analyses were performed on healthy and diseased *C. chinensis* rhizomes. This study found that root rot can lead to the significant reduction of medicinal components of Coptis, including thaliotrine, columbamine, epiberberin, coptisine, palmatine chloride, and berberine, affecting its efficacy quality. In the present study, *Diaporthe eres*, Fusarium *avenaceum*, and Fusarium solani were identified as the main pathogens causing root rot in *C. chinensis*. At the same time, the genes in phenylpropanoid biosynthesis, plant hormone signal transduction, plant-pathogen interaction, and alkaloid synthesis pathways were involved in the regulation of root rot resistance and medicinal component synthesis. In addition, harmful pathogens (*D. eres*, *F. avenaceum* and F. solani) also induce the expression of related genes in *C. chinensis* root tissues to reduce active medicinal ingredients. These results provide insights into the root rot tolerance study and pave the way for process disease resistance breeding and quality production of *C. chinensis*.

**IMPORTANCE** Root rot disease significantly reduces the medicinal quality of *Coptis chinensis*. In the present study, results found that the *C. chinensis* fibrous and taproot have different tactics in response to rot pathogen infection. *Diaporthe eres*, Fusarium *avenaceum*, and Fusarium solani were isolated and identified to cause different degrees of *C. chinensis* root rot. These results are helpful for researchers to further explore the mechanism of resistance to rhizoma Coptis root rot.

## INTRODUCTION

*Coptis chinensis* Franch (2n = 2× = 18, “Weilian” in Chinese), belonging to the *Ranunculaceae*, is a native perennial medicinal herb plant as a traditional Chinese medicine for thousands of years (1–3). *C. chinensis* prefers an adumbral, moist, and cold climate growing environment at 500 to 2,000 m elevation (4, 5), and is primarily grown in China's Chongqing (eastern), Hubei (western), Hunan (northern), and Guizhou provinces. In China, Chongqing (Shizhu County) and Hubei (Lichuan City) are the main regions for product processing of *C. chinensis* (6). The *C. chinensis* plant has a high therapeutic value and is widely used medically due to its yellow and branched rhizomes (5, 6). The most abundant active components of *C. chinensis* are alkaloids beneficial to human health, including berberine, palmatine chloride, coptisine, jatrorrhizine, columbamine, thaliotrine, and epiberberine. Many studies have illustrated that these alkaloid compounds have a wide range of pharmacological activities, such as antiinflammatory, antimicrobial activity, antidiabetic, antiatherosclerotic, antiviral, antitumor effects, dispelling dampness, and detoxification agents (7–9). However, the alkaloid content in the rhizomes of *C. chinensis* is easily inhibited by root rot disease, and even directly causes the death of the whole plant.

Root rot is a severe plant-pathogenic fungus disease that generally causes the aboveground part of the plant to wilt and the whole plant to die, which is also the main factor constraining medicinal herb rhizome yield, the content of medicinal ingredients (quality), and marketability in China (10–12). In *C. chinensis*, root rot will seriously affect the use of medicinal effects and become a major threat to its production and industry development (13). The brown speckle or progressive necrosis that emerges in the rhizomes or fibrous roots can cause mortality of the infected whole plants (11). A previous study has found that the annual infection rate of root rot disease is 10 to 20% in the largest production region of *C. chinensis* in Chongqing (Shizhu). In severe cases, the root rot disease infection rate can even reach 60 to 90%, causing no harvest of *C. chinensis* (13). The root rot disease (infected plant) can be introduced by transplant and field production systems harming agricultural production. At the present time, there are almost no reports on the resistance mechanism of *C. chinensis* root rot and no environmentally friendly way to eradicate plant root rot. Therefore, it is a necessary procedure to study the origin, pathogenesis, and defense mechanisms of plant root rot disease to prevent and control the occurrence of root rot.

Studies have found a correlation between root rot disease and continuous cropping, which always causes a decrease in herb plant growth and considerable active pharmaceutical ingredient yield loss (14, 15). Previous research has demonstrated that long-term continuous cultivation of the same crops for many years changes the microbial community of plants in the rhizosphere soil (5, 15, 16), which further contributes to the continuous cropping obstacles and root rot occurring (17). However, little information has been acquired about the specific response mechanism of continuously cropping soil microorganisms to the root rot of medicinal plants. Therefore, in order to meet the needs for production and medicinal quality of *C. chinensis*, it is necessary to study the relationship between root rot pathogenesis and continuous cropping obstacles.

In order to explore the pathogenic causes and response mechanisms of *C. chinensis* root rot, transcriptome and microbiome analyses were conducted on healthy and diseased *C. chinensis* rhizomes to reveal the potential mechanisms, defense system, and root rot pathogens. Therefore, the aim of this study was (i) to explore the effect of *C. chinensis* root rot on medicinal quality (alkaloid content), (ii) to elucidate the relationship between root rot disease and gene expression profile, (iii) to isolate the microbial community structure and pathogens causing root rot of *C. chinensis*, (iv) to identify the candidate genes involved in the disease resistance of *C. chinensis*, and (v) to reveal the characteristics and potential pathogenic mechanism. These findings can help to advance the process of disease resistance breeding and quality production of *C. chinensis* by providing insights into the underlying molecular mechanisms and microorganisms for rhizome rot tolerance.

## RESULTS

### The content of active ingredients changed in root rot *C. chinensis*.

**FIG 1:**
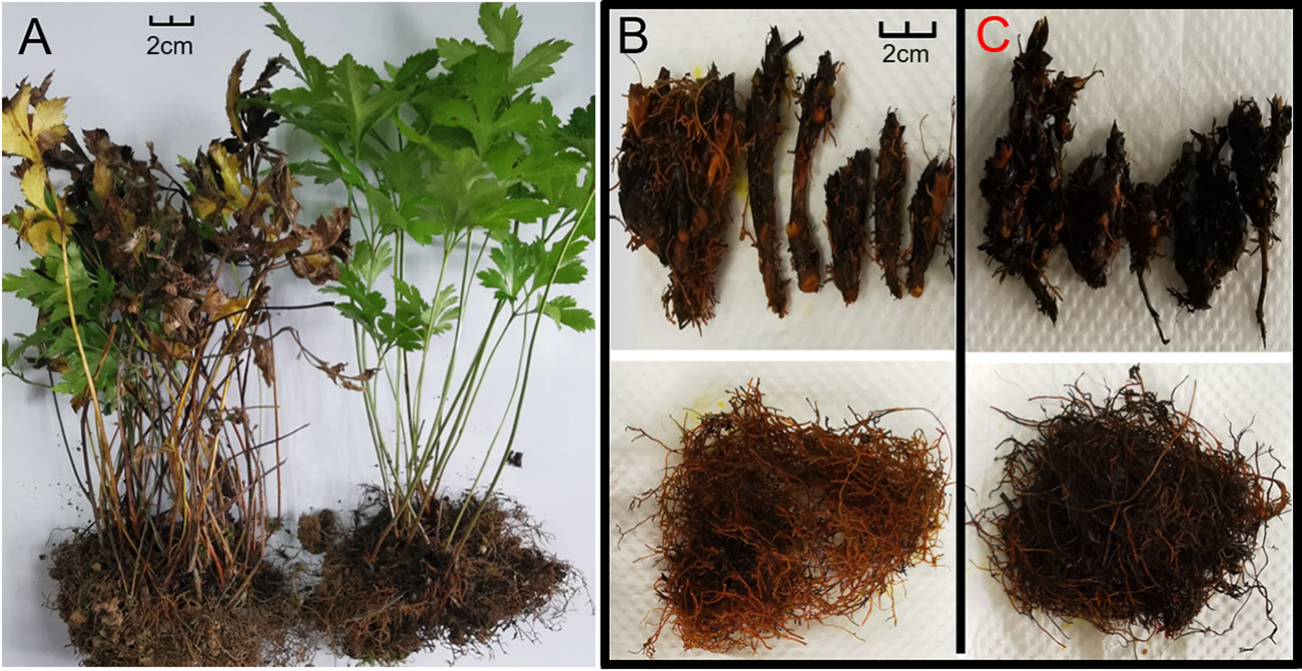
Phenotypes of root rot and healthy *Coptis chinensis*. (A) Overall phenotype of 3-year-old root rot and healthy *C. chinensis* plants. (B) Primary and fibrous roots of healthy Coptis. (C) Primary root and fibrous root of diseased Coptis. The scale label in the figure represents 2 cm.

**FIG 2:**
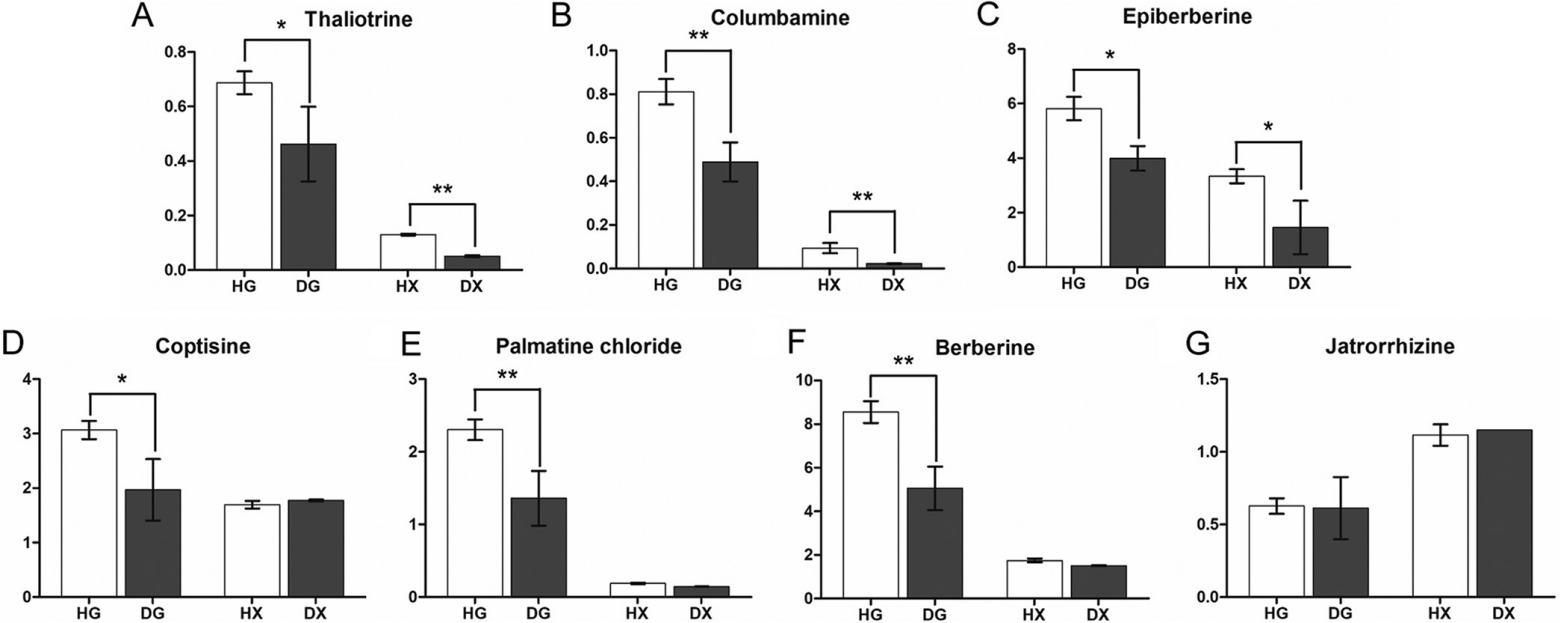
Determination of main medicinal active ingredients in healthy and root rot Coptis root (primary root and fibrous root). (A) Thaliotrine; (B) Columbamine; (C) Epiberberine; (D) Coptisine; (E) Palmatine chloride; (F) Berberine; (G) Jatrorrhizine. In the figure, HG and HX represent the primary root and fibrous root of healthy Coptis, respectively. DG and DX represent the primary root and fibrous root of Coptis, respectively. In each bar chart *, *P* < 0.05; **, *P* < 0.01.

The content of the main active components was evaluated in healthy and diseased (root rot) roots of *C. chinensis* to systematically evaluate the influence of rhizome decay on root growth and medicinal components accumulation (Fig. 1). The main medicinal components in *C. chinensis* include thaliotrine, columbamine, epiberberin, coptisine, palmatine chloride, berberin, and jatrorrhizine. The results showed that Berberin had the highest content in the healthy roots (primary root HG and fibrous root HX) of *C. chinensis*, followed by epiberberin, coptisine, and palmatine chloride (Fig. 2). In addition, the content of these medicinal active ingredients in the main rhizome of *C. chinensis* was significantly higher than that in fibrous roots (Fig. 2), which was consistent with previous reports. However, root rot significantly reduced the content of important medicinal active ingredients in *C. chinensis* in both the diseased taproot (DG) and diseased fibrous roots (DX), which include berberin, thaliotrine, columbamine, epiberberin, coptisine, and palmatine chloride, thus seriously affecting the quality of *C. chinensis*. Compared with HG, in DG, thaliotrine, columbamine, epiberberin, coptisine, palmatine chloride, and berberin were all decreased significantly by 1.49 times, 1.66 times, 1.46 times, 1.55 times, 1.69 times, and 1.69 times, respectively (Fig. 2). Compared with HX, the content of these medicinal substances in DX was also significantly reduced (Fig. 2), while the content of jatrorrhizine had no significant change in HG, DG, HX and DX (Fig. 2G). Based on these results, the present study found that root rot caused serious harm to the medicinal quality of *C. chinensis*. At the same time, the results suggested that harmful pathogens could induce the expression of related genes in *C. chinensis* root cells or tissues to regulate the synthesis or reduction of active medicinal compounds. Therefore, to investigate the pathogenic mechanism of root rot in *C. chinensis*, this study analyzed the transcriptome (gene expression profile) results of taproot and fibrous roots with root rot disease infection.

### Transcriptome analysis of *C. chinensis* in healthy and disease roots.

**FIG 3:**
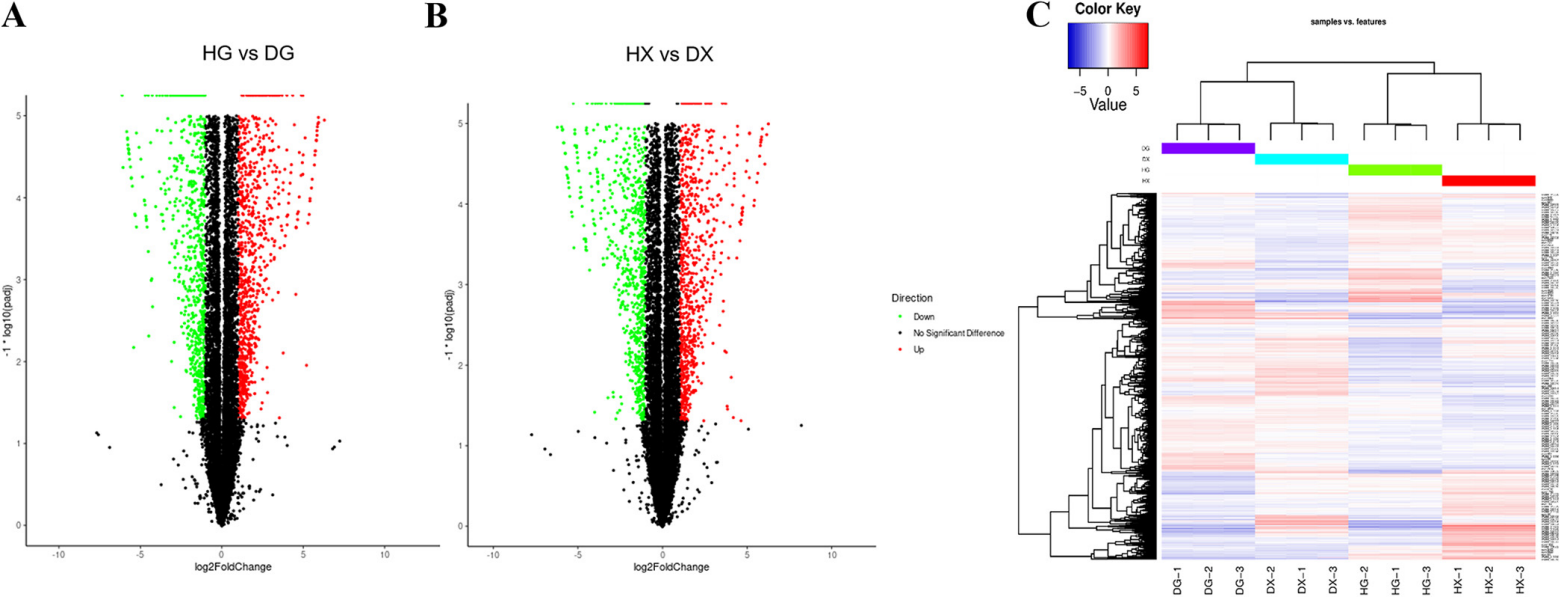
Volcano plots and hierarchical clustering analysis of differentially expressed genes (DEGs) in root rot and healthy Coptis. (A) DEGs analysis of HG versus DG comparison group. (B) DEGs analysis in HX versus DX comparison group. (C) Hierarchical clustering and gene expression pattern analysis of all DEGs. In the figure, HG and HX represent the primary root and fibrous root of healthy Coptis, respectively. DG and DX represent the primary root and fibrous root of Coptis, respectively. Red color represents gene expression upregulated, blue color represents downregulated gene expression.

This experiment used the software DESeq2 (version 1.26.0), screening threshold of padj < 0.05, and |log2FoldChange| >1 to screen differentially expressed genes (DEGs) in taproot and fibrous roots. In HG versus DG, a total of 5397 DEGs were identified, including 2,823 upregulated genes and 2,574 downregulated genes (Fig. 3A). A total of 4909 DEGs were screened in HX versus DX, of which 2,374 were upregulated and 2,535 were downregulated (Fig. 3B). The results suggested that gene expression was significantly affected by root rot disease infection, and thus the growth and development of the whole plant were affected. Before detailed analysis, cluster analysis was performed on all differentially expressed genes (Fig. 3C). The results showed that the gene expression profile of the rhizome of *C. chinensis* changed significantly between healthy and diseased roots. The healthy samples (pathogen infection before, HG, HX) clustered into one branch, and the disease root samples (pathogen infection after, DG, DX) clustered into another branch (Fig. 3C). The results suggested that the differences in DEG expression patterns in different tissue parts of *C. chinensis* were less than those in healthy and diseased roots, which also provided reliable information and a basis for the subsequent identification of DEGs related to root rot disease.

### KEGG analysis of DEGs in healthy and root rot roots of *C. chinensis*.

KEGG enrichment analysis was performed for HX versus DX and HG versus DG. The results showed that in HX versus DX, gene enrichment pathways mainly included plant-pathogen interaction, phenylpropanoid biosynthesis, and starch and sucrose metabolism (Fig. S1A). In HG versus DG, gene enrichment pathways mainly include: phenylpropanoid biosynthesis, monoterpenoid biosynthesis, and alpha-linolenic acid metabolism (Fig. S1B). And in HG VS DG, the MAPK signaling pathway and the plant hormone signal transduction pathway are also enriched, but not significantly (Fig. S1B). Therefore, the results suggest that these are the main pathways responding to root rot infection in *C. chinensis*, and this study will analyze these pathways in detail.

### The DEGs related to phenylpropanoid and plant hormone synthesis in *C. chinensis*.

**FIG 4:**
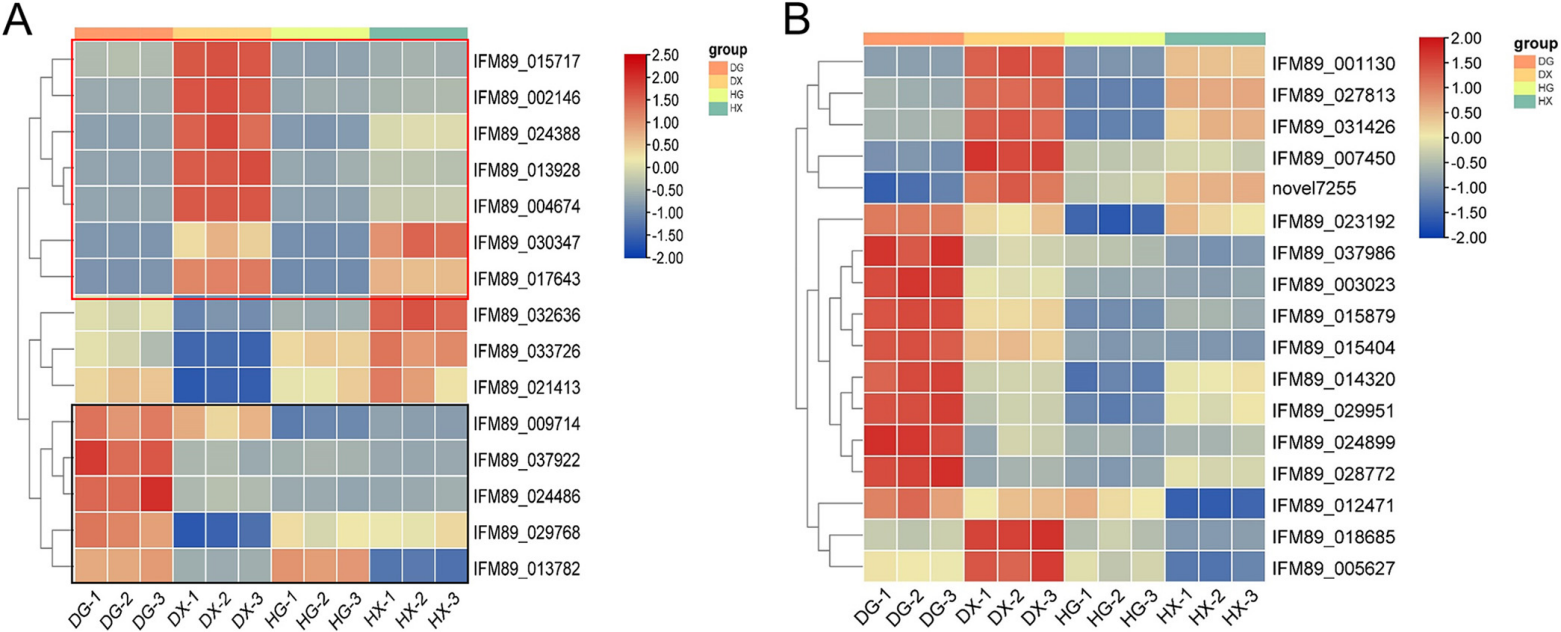
Analysis of DEGs expression patterns associated with Coptis root rot. (A) Analysis of the expression pattern of DEGs in the Phenylpropanoid biosynthesis pathway involved in Coptis resistance to root rot. (B) DEGs enriched in plant hormone signal transduction pathway to involved in response to root rot. In the figure, HG and HX represent the primary root and fibrous root of healthy Coptis, respectively. DG and DX represent the primary root and fibrous root of Coptis, respectively. Red color represents gene expression upregulated, blue color represents downregulated gene expression.

The phenylpropanoid biosynthesis pathway was significantly enriched in the HX versus DX and HG versus DG comparison groups. Therefore, this study first analyzed changes in gene expression levels in this pathway. The results showed that 15 DEGs were identified for HX versus DX and HG versus DG, among which 7 DEGs were significantly upregulated in DX and 5 DEGs were upregulated in DG (Fig. 4A). The results illustrated that the fibrous roots and taproot of *C. chinensis* showed different stress responses after infection by the root rot pathogen. Detailed analysis revealed that the DEGs were upregulated in DX, including coniferyl-aldehyde dehydrogenase (REF1), catechol-O-methyltransferase (COMT), bglB beta-glucosidase, and cinnamyl-alcohol dehydrogenase. The upregulated DEGs in DG include EC:1.11.1.7, cinnamyl-alcohol dehydrogenase (CAD), 4-coumarate-CoA ligase (4CL), and beta-glucosidase.

The present study then analyzed changes in gene expression levels in plant hormone signal transduction pathways. A total of 17 related differentially expressed genes were identified, of which 10 DEGs were highly expressed in DG and the other 7 DEGs were highly expressed in DX (Fig. 4B). The results showed that there were significant differences in key regulatory DEGs in response to root rot pathogen infection in different tissues of *C. chinensis* (taproot and fibrous root). However, on the whole, these DEGs mainly showed upregulated expression patterns. These genes involved in root rot disease mainly include: AHK2_3_4 (IFM89_003023), SNRK2 (IFM89_014082, IFM89_015404, IFM89_037986), ABF (IFM89_025031), ETR (IFM89_023192), CTR1 (IFM89_029951), EIN2 (IFM89_012471), etc.

### The DEGs related to plant pathogen interaction and alkaloid synthesis in *C. chinensis*.

**FIG 5:**
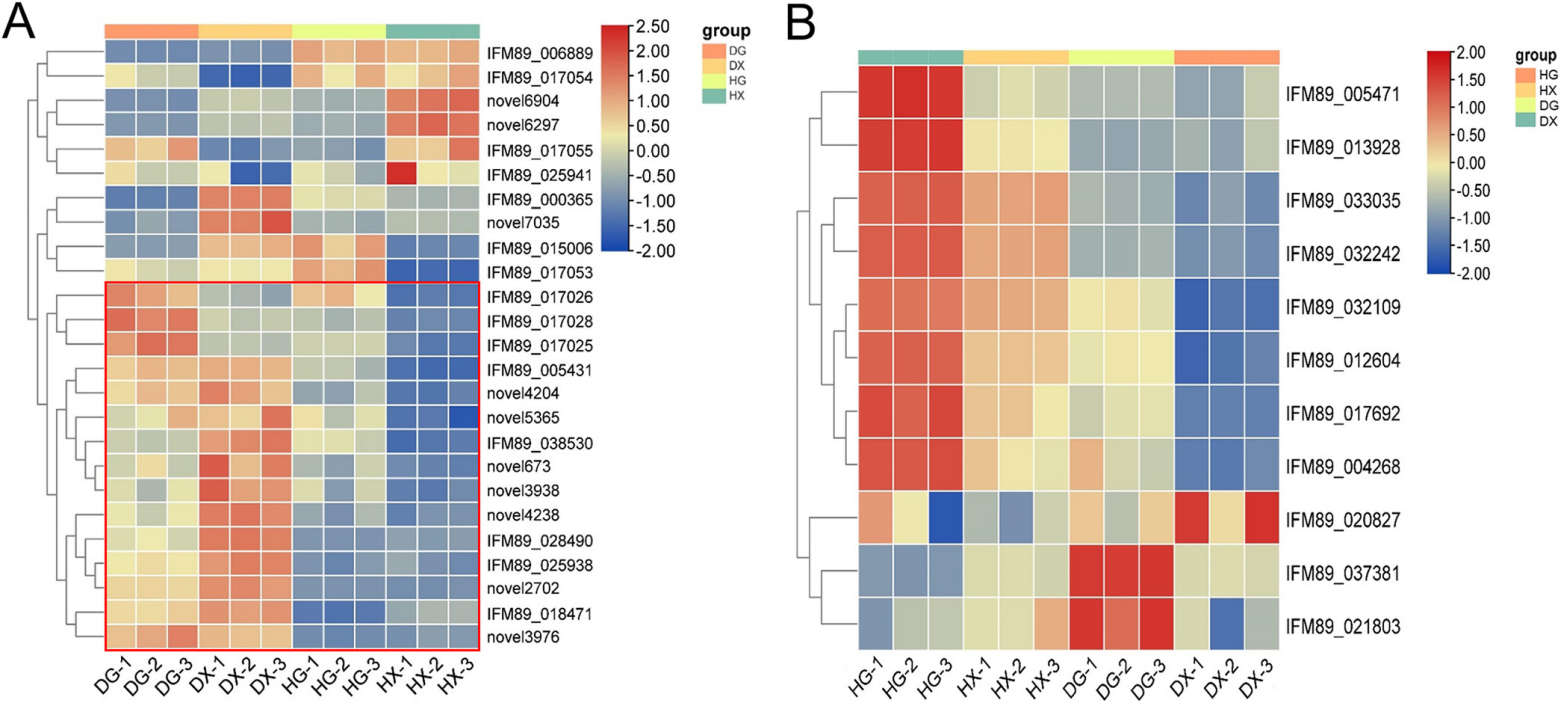
Analysis of DEGs expression patterns associated with Coptis responding to root rot pathogens. (A) DEGs enriched in the plant-pathogen interaction to involved in response to root rot. (B) Expression pattern analysis of DEGs enriched in alkaloid synthesis pathway. In the figure, HG and HX represent the primary root and fibrous root of healthy Coptis, respectively. DG and DX represent the primary root and fibrous root of Coptis, respectively. Red color represents gene expression upregulated, blue color represents downregulated gene expression.

In this study, the changes of differentially expressed genes in the plant-pathogen interaction pathway were analyzed. A total of 47 DEGs related to plant-pathogen interaction were screened out from the HX versus DX and HG versus DG comparison groups, and 25 genes were screened out from those whose gene expression levels were all lower than 10 (Fig. 5A). The results showed that compared with healthy *C. chinensis* (HG, HX), 15 DEGs were significantly upregulated in diseased *C. chinensis* (DG, DX) (Fig. 5A). And the expression level of DEGs was the most significant in the fibrous roots of *C. chinensis* (HX versus DX), which was consistent with the result of the phenotypic differences. Therefore, this study speculated that these DEGs were involved in the regulation of root rot disease in *C. chinensis*, and a detailed analysis revealed that certain genes were mainly involved in root rot, such as RPS2 (IFM89_017028, novel4238, novel2702, novel5365, novel4204, novel3976, IFM89_028490, novel673, novel3938, IFM89_017025, IFM89_017026, and IFM89_025938), SGT1 (IFM89_038530), and RIN4 (IFM89_018471).

In addition, this study also analyzed the expression profile of DEGs involved in alkaloid synthesis in *C. chinensis*. The results showed that a total of 11 DEGs involved in the synthesis of alkaloids had significant expression variation. Among them, compared with DG and DX, there are 8 DEGs significantly upregulated in HG and HX, which include IFM89-005471, IFM89_013928, IFM89_033035, IFM89_032242, IFM89_032109, IFM89_012604, IFM89_017692, and IFM89_004268 (Fig. 5B). These results were consistent with the previous results of alkaloid detection (active medicinal ingredients). Furthermore, the expression levels of IFM89_037381 and IFM89_021803 were HG < DG, HX > DX (Fig. 5B). Therefore, the results of this experiment indicated that the rhizobium rot pathogen regulated or affected the expression level of alkaloid synthesis related genes in the root tissues of *C. chinensis*, affecting the alkaloid content and medicinal quality of *C. chinensis*.

### Analysis of soil microbial diversity in rhizosphere of *C. chinensis*.

In order to explore the mechanism of root rot in *C. chinensis*, as well as the effect of root rot on the underground microflora of *C. chinensis*, and to identify potential pathogen organisms. This experiment also combined soil nutrient changes with rhizosphere microbial diversity detection for health and root rot *C. chinensis*. The bacterial amplicons in the rhizosphere soil of healthy plants (10 plants), diseased plants (10 plants), and the soil without *C. chinensis* cultivation were sequenced. A total of 153493 high-quality reads were obtained, and then OUT cluster analysis was performed on these data. At the same time, to visualize the similarity and difference of microbial communities between healthy and diseased *C. chinensis* rhizosphere soil samples, this study analyzed the bacterial α diversity index (Fig. S2A). The results showed that the bacterial abundance of diseased *C. chinensis* was significantly lower than that of unplanted and healthy *C. chinensis* (*P* < 0.05) in rhizosphere soil, and the diversity of the bacterial population was also significantly lower than that of unplanted *C. chinensis* soil. The abundance of bacteria in never planted soil samples was significantly higher than that of *C. chinensis* planted continuously soil (*P* < 0.05). These results indicated that root rot significantly affected the rhizosphere bacterial community of *C. chinensis*, and thus had a serious impact on soil bacterial and microbial diversity. The planting of *C. chinensis* (continuous cropping) reduced the diversity of soil microorganisms. As a result, these findings suggested that continuous cultivation of *C. chinensis* could result in continuous cropping disorder and root rot.

The further analysis results showed that, compared with *C. chinensis* that had not been planted in soil (N. RMS3), the cultivation of *C. chinensis* (H. RMS3 and D. RMS3) significantly increased the relative abundance of Proteobacteria, Actinobacteria, and Crenarchaeota (Fig. S2B). Firmicutes and Gemmatimonadetes differences were not significant between healthy and diseased rhizosphere soil. The cultivation of *C. chinensis* also significantly reduced the relative abundance of Acidobacteria and Planctomycetes (*P* < 0.05). Compared with H. RMS3, the abundance of Firmicutes and Crenarchaeota was significantly reduced in D. RMS3, while the relative abundance of Gemmatimonadetes was significantly increased (*P* < 0.05) (Fig. S2B).

### Analysis of microbial abundance in rhizosphere soil of *C. chinensis*.

**FIG 6:**
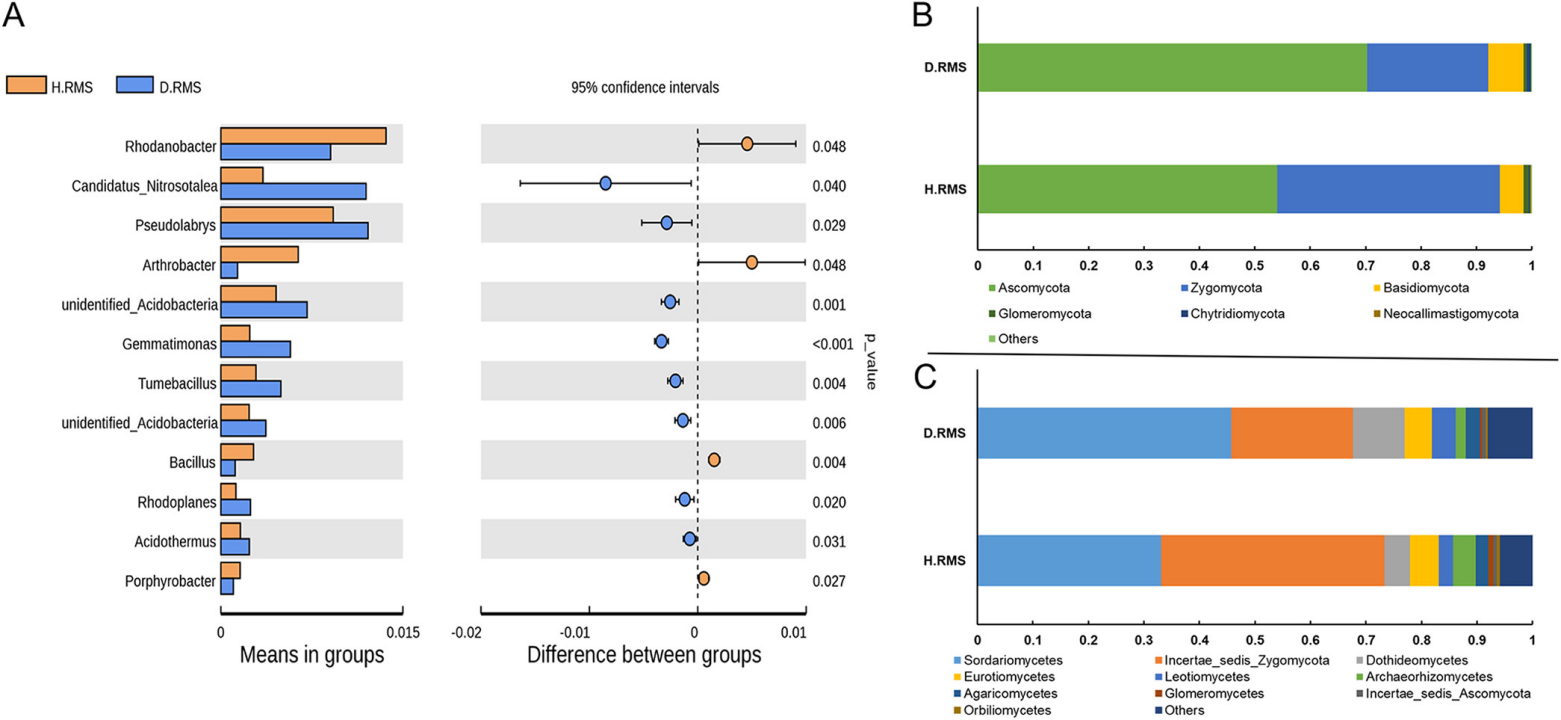
(A) Significance test (Wilcoxon rank sum test) between healthy and diseased rhizosphere soil bacterial and fungal community groups. (B) Composition of the different fungal phyla (top 10) in *C. chinensis* rhizosphere soil. (C) The top 10 fungal genera community abundance of the healthy and diseased rhizosphere soil. H. RMS3 and D. RMS3 were cultivation of health and root rot *C. chinensis* soil, respectively.

In order to find the differences between healthy and diseased *C. chinensis* rhizosphere soil microbial species at the genus level, this study screened out significant differences in microbial species by T-test (*P* < 0.05). The results revealed a significant decrease in the abundance of Rhodanobacter, Arthrobacter, and Bacillus, while the abundance of Candidatus Nitrosotalea, Pseudolabrys, and Gemmatimonas increased (Fig. 6A). Therefore, the result speculated that root rot can significantly impact on the above microbial communities, which caused the root system of *C. chinensis* to be infected and limit plant growth.

This study then analyzed the composition, richness and diversity of fungi in the diseased and healthy rhizomes of *C. chinensis* rhizosphere soils. In these two types of samples of *C. chinensis* rhizosphere soil, the dominant fungi communities are Ascomycota, Zygomycota, and Basidiomycota (Fig. 6B). Furthermore, there was a significant difference in fungi abundance class level in healthy and diseased *C. chinensis* rhizosphere soil (Fig. 6C). In D. RMS, the abundance of *Dothideomycetes*, *Sordariomycetes*, and *Leotiomycetes* was significantly higher than that of H. RMS, while the abundance of Incertae sedis Zygomycota, Eurotiomycetes, and Archaeorhizomycetes were significantly lower than that of H. RMS.

### The relationship between pathogenic of root rot and soil nutrients.

**FIG 7:**
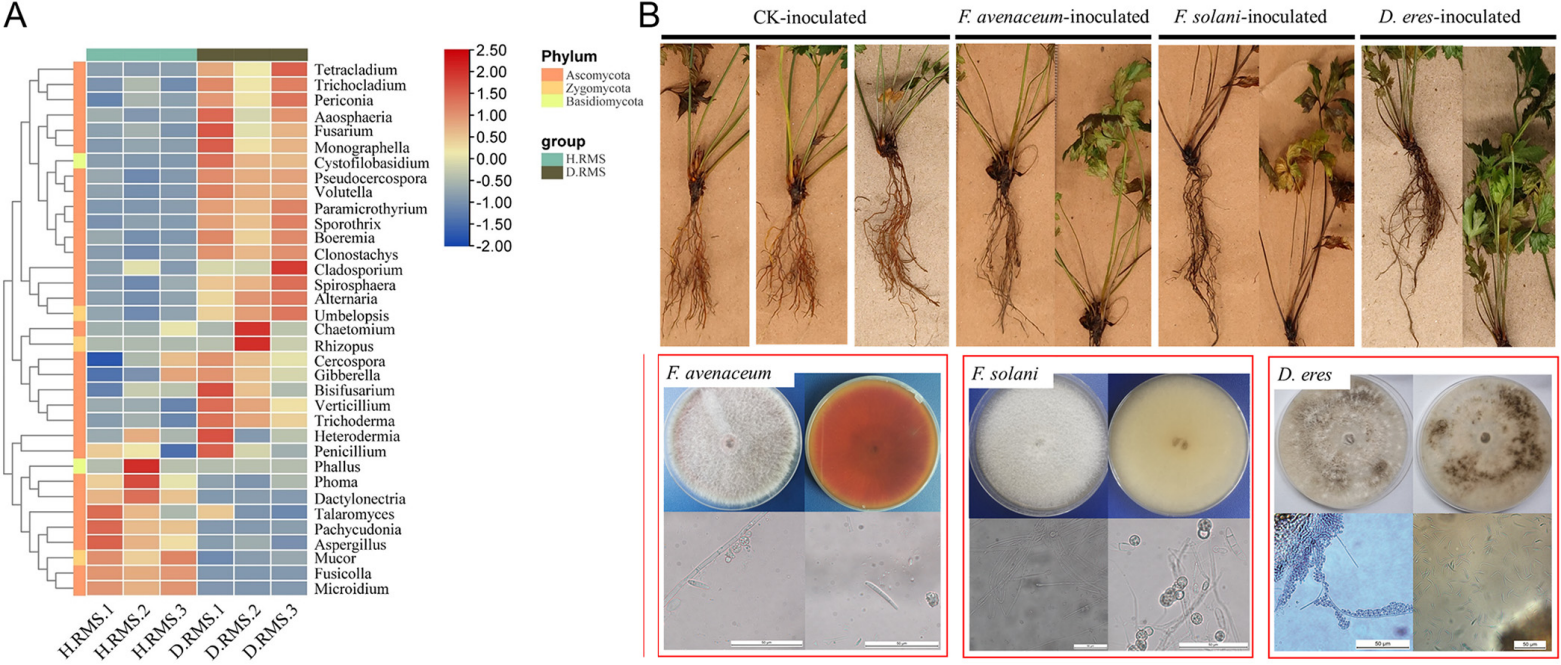
(A) Changes in the abundance of various pathogens related to root rot in rhizosphere soil of Coptis with different treatment groups. (B) Isolation and validation of the three main pathogens to cause Coptis root rot. H. RMS3 and D. RMS3 were cultivation of health and root rot *C. chinensis* soil, respectively.

In the present study, species annotation and abundance information of bacterial microorganisms were obtained from all samples at the genus level. The top 35 genera were chosen by analyzing their abundance in the three groups of rhizosphere soil samples in order to identify potential pathogens causing rhizoma Coptis root rot. The results showed the abundance of Fusarium, *Aaosphaeria*, *Trichocladium*, *Clonostachys*, *Volutella*, and *Monographella* in diseased rhizosphere soil samples (D. RMS) was higher than that in healthy rhizosphere soil samples (H. RMS). The abundance of *Mucor*, *Microidium*, *Phoma*, *Fusicolla*, *Phallus* was lower than that of healthy rhizosphere soil samples (H. RMS) (Fig. 7A).

This experiment also measured PH, organic carbon, alkali-hydrolyzed nitrogen, available phosphorus and available potassium in the rhizosphere soil of healthy and diseased Coptis to analyze the influence of soil nutrients on root rot disease (Table 1). The results showed that the contents of organic carbon and alkali-hydrolyzed nitrogen in the rhizosphere soil of diseased Coptis were significantly lower than those of healthy plants, while the contents of PH, available phosphorus and available potassium in the soil were higher than those of healthy plants. Spearman’s analysis showed that the abundance of Fusarium fungi was significantly positively correlated with PH and available phosphorus content, while negatively correlated with alkali-hydrolyzed nitrogen content (*P* < 0.05). Therefore, the results suggest that Fusarium is the main pathogen causing Coptis root rot. Subsequently, this study analyzed the dominant strains of Fusarium SPP. and found that Fusarium *avenaceum* and Fusarium solani were the dominant strains with 0.575% and 0.779% relative content in healthy and diseased plant rhizosphere soil, respectively (Fig. 7B). In addition, another pathogen not belonging to the genus Fusarium, *Diaporthe eres* were identified, which may also be one of the main pathogens causing Coptis root rot. In order to verify the pathogenicity of the root rot caused by these microbes, this study inoculated these fungi into the root of Coptis for verification (Fig. 7B). The results showed that *Diaporthe eres*, Fusarium *avenaceum* and Fusarium solani caused different degrees of root rot in *C. chinensis*.

**TABLE 1:** The soil nutrients detection in rhizosphere soil of healthy and diseased Coptis^*a*^

Group	PH	Organic carbon (g/kg)	Alkali-hydrolyzable nitrogen (mg/kg)	Available P (mg/kg)	Available K (mg/kg)
H.RMS	5.6	7.47	179.4	135.68	394.78
D.RMS	5.78	6.3	137.87	140.89	961.43

a H. RMS3 and D. RMS3 were cultivation of health and root rot *C. chinensis* soil, respectively.

## DISCUSSION

*C. chinensis* rhizome (with an extremely bitter taste) has plentiful pharmacological activities that help detoxify and prevent disease progression (18). The active ingredients of *C. chinensis* (mainly alkaloids) also have antibacterial and anti-inflammatory effects, and can treat jaundice, diarrhea, vomiting, dysentery, clearing heat antitoxicant, toothache, abdominal distension, high fever coma, high cholesterol, diabetes, eczema, etc. (15, 19–21). However, root rot is the main limiting factor affecting the growth, medicinal quality, and production of *C. chinensis*. The species of root rot pathogen and the mechanisms of pathogen infection and plant resistance are still not clear in *C. chinensis*. Therefore, in this study, the gene expression profiles and rhizosphere soil microorganisms in healthy and root rot disease of *C. chinensis* were analyzed and verified. The main pathogens and the key candidate genes for responding to root rot were identified that provide insights and a theoretical basis for the study of the rhizome of *C. chinensis* resistance to root rot disease.

More than 30 alkaloids have been extracted and identified in *C. chinensis*. Among them, the main components of these alkaloids include berberine, epiberberine, columbamine, coptisine, palmatine chloride, thaliotrine, and jatrorrhizine (22, 23), and the content and type of these alkaloids determines the quality of *C. chinensis*. In this study, the major alkaloid components in the taproots and fibrous roots of healthy and diseased *C. chinensis* were also determined. The results showed that the alkaloid content of the taproot was significantly higher than that of the fibrous root in *C. chinensis*. Compared with healthy roots (taproots and fibrous roots), the content of alkaloids, such as berberine, epiberberine, columbamine, coptisine, palmatine chloride, and thaliotrine, were significantly reduced in root rot. Furthermore, the expression profiles of alkaloid synthesis-related DEGs matched the variation trend of these alkaloids' accumulation pattern. These results indicated that root rot disease seriously affected the medicinal quality of *C. chinensis*.

Berberine is the main pharmacologically active component of *C. chinensis* is involved in the regulation of P450 gene expression, which can also improve the chronic inflammatory state of type 2 diabetes mellitus, change or stimulate the transformation of brown adipose tissue to white, promote energy metabolism, improve insulin resistance and improve glucose metabolism (24–26). In the present study, the regulated genes, including cytochrome P450 (IFM89_017692), coclaurine N-methyltransferase (IFM89_033035), S-norcoclaurine synthase (IFM89_032242, IFM89_032109, and IFM89_012604), cytoplasmic tRNA 2-thiolation protein (IFM89_004268), and Protein-l-isoaspartate (d-aspartate) O-methyltransferase (IFM89_005471) were identification significantly downregulated by root rot pathogen infection in *C. chinensis*. Previous studies have shown that certain enzyme genes such as P450, methyltransferase, dehydrogenase, acetyltransferase, and glycosyltransferase (27) are catalyzed by central precursors (derivatives of the amino acid tryptophan) to synthesize alkaloids. Therefore, the gene expression variation regulated berberine biosynthesis in the root rot diseased plant, causing the berberine content to decrease and seriously damaging the medicinal value and efficacy of *C. chinensis*.

### Root rot associated pathogen identification of *C. chinensis*.

*Coptis chinensis* is a traditional Chinese medicine plant that the medicinal part is tuberous. However, similar to other tuberous medicinal plant, root rot is the main disease that harms the production and quality of *C. chinensis*. Previous studies have illustrated that plant metabolites and microflora contribute to host plant defense against pathogens (11, 28, 29). Recently, there has been increasing evidence that root/rhizosphere microbiota are directly or indirectly involved in host plant metabolic processes by regulating host secondary or specialized metabolism (30). The results of the present study showed that *Diaporthe eres*, Fusarium *avenaceum*, and Fusarium solani were the dominant pathogens in the rhizosphere microbial community to cause root rot in *C. chinensis*. Among them, Fusarium *avenaceum* and Fusarium solani were consistent with the previous study's findings that both were pathogens closely related to *C. chinensis* root rot (11). And different from previous studies, the present study found that the abundance of Fusarium solani in healthy and diseased Coptis rhizosphere soils was significantly lower than that of Fusarium *avenaceum*. This result suggested that *F. avenaceum* may be the most important pathogen causing rhizoma Coptis rot.

In addition, *Diaporthe eres*, another dominant pathogen of non-fusarium, was isolated from the rhizome of *C. chinensis* for the first time. *D. eres*, belonging to the genus *Hemipychomycota*, has been recently isolated and reported in many plants. *D. eres* causes hazelnut canker disease of fruit trees and nut crops, which causes black spots, brown spots, necrotic spots, visible mildew spots on the kernel surface, and fruit interior discoloration at the hazelnut tip (31, 32). In 2007, the pathogen *D. eres* was isolated from butternut, which causes black lesions on the branches and stems of living saplings (33). However, in the present study, *D. eres* was verified as one of the main pathogens to cause root rot in Coptis, which was also reported as mainly harmful to newly established saplings (forming 2 to 5 cm oval spots on the treetops). The spots turn dark brown at the bottom and spread upward. Furthermore, *D. eres* is a minor pathogen of certain woody plants that can cause plant death in plants including peaches (34), pears (35), blueberries (36), and grapes (37) and in the branches of *Corylus avellana* (38). The present study found that after inoculation with *D. eres*, the rhizome of healthy *C. chinensis* gradually spread outward from a small necrotic spot to the whole tuber. Finally, the root rot of *C. chinensis* was aggravated and deteriorated, and its yield was seriously reduced.

### Effects of phenylpropanoid and phytohormones on root rot resistance.

Rhizome rot is a severe pathogen disease that can cause great damage and poses a severe threat to *C. chinensis* development. Pathogens usually penetrate the root xylem through the cortex and secrete toxins that cause leaf chlorosis and symptoms of intervein necrosis, leading to premature plant death (39, 40). The plant produces a corresponding immune system response to pathogen infection. Therefore, this study found that the expression levels of DEGs related to plant immunity (resistance) in *C. chinensis* roots changed significantly after pathogen infection. Pathogens have different strategies and extremely complex mechanisms for infecting and colonizing plants, also specifically targeting different hosts (41, 42). However, the gene expression profile of Coptis root tissue responding to pathogens has not been reported. Therefore, in addition to identifying the main pathogens causing Coptis root rot, this study also analyzed the mechanism of plant response to pathogen infection and identified key candidate genes.

Genes significantly enriched in phenylpropanoid biosynthesis and plant hormone signal transduction pathways determine plant resistance to abiotic and biotic stresses (43). The present study found that the expression levels of key enrichment DEGs were significantly different in plant-pathogen interaction, phenylpropanoid biosynthesis, and plant hormone signal transduction pathways in healthy and diseased Coptis root by RNA-Seq profile analysis. Previous studies have shown that phytohormone biosynthesis can enhance plant resistance to pests by activating the phenylpropanoid pathway (44). Therefore, the biological pathways involved in hormone signal transduction were responsible for plant resistance to diseases (45). Plant resistance was associated with related genes in plant hormone signal transduction pathways, including AHK2_3_4, SNRK2, ABF, ETR, CTR1, and EIN2; all were significantly higher expressed in diseased Coptis in response to pathogen infection. Furthermore, in the phenylpropanoid biosynthesis pathway, the DEGs EC:1.11.1.7, cinnamyl alcohol dehydrogenase (CAD), 4CL, and beta-glucosidase were significantly higher expressed in diseased Coptis. Among them, CAD, POD, β-glucosidase, and PAL play a crucial role in plant defense response and participate in plant resistance to pathogens (46). In addition, this study also found that RPS2 gene family members in the plant-pathogen interaction pathway in Coptis were significantly upregulated after root rot pathogen infection, suggesting that pathogen infection caused resistance responses in the rhizome of Coptis (47). These genes might regulate the synthesis of related small molecules in plant tissues that play an important role in Coptis defense against pathogen attack by fine-tuning plant hormones, phenylpropanoid, plant-pathogen interaction, and disease resistance mechanisms.

### Conclusion.

Root rot has caused irreversible damage and a great loss to the medicinal quality and production yield of Coptis. Therefore, high-throughput sequencing and metabolic profiling were conducted to systematically study the mechanism of rhizome rot on Coptis growth and medicinal components. The results showed that root rot notably reduced the content of the main active components (thaliotrine, columbamine, epiberberin, coptisine, palmatine chloride, berberin, and jatrorrhizine) in *C. chinensis*. Eleven key genes related to alkaloid synthesis were identified in *C. chinensis*. The rhizosphere microbial community of copitis was significantly affected by root rot disease and continuous cropping reduced the diversity of soil microorganisms. This study isolated and verified the main pathogens that cause different degrees of copitis root rot, namely, *Diaporthe eres*, Fusarium *avenaceum*, and Fusarium solani. In addition, genes related to copitis response to root rot pathogens, such as REF1, COMT, beta-glucosidase, CAD, 4CL, cinnamyl-alcohol dehydrogenase, SNRK2, ABF, ETR, RPS2, and RIN4 in phenylpropanoid biosynthesis, plant hormone signal transduction and plant-pathogen interaction pathways, were also identified, respectively. The regulation of these genes is essential for Coptis in response to root rot pathogen infection. Therefore, genetic engineering techniques can be used to regulate the expression or silencing of these genes to make Coptis plants resistant to root rot pathogens. The isolation of pathogens is also helpful for researchers to further develop targeted drugs to overcome the occurrence of Coptis root rot.

## MATERIALS AND METHODS

### Plant material.

Rhizomes (taproot and fibrous roots) of healthy and root rot 3-year-old Coptis were used in this study. Samples were collected from a GAP planting base with an altitude of 1,667 m, 1,200 mm of annual precipitation, and an annual average temperature of 16°C in Chongqing, Shizhu (108°26 'E, 29°59' N) during the prevalence of Coptis root-rotted disease (August). The healthy and root rot plant roots were dug out and washed with clean water (more than five times) to remove surface soil (Fig. 1A). Then, the cleaned root was separated from the taproot and fibrous root (Fig. 1B, C), and put into a centrifuge tube. At least 10 Coptis plants were collected from each treatment group and mixed as one sample, which was repeated three times. All Coptis root samples were quickly frozen in a liquid nitrogen tank and brought back to the laboratory for storage at –80°C refrigerators to prepare for subsequent DNA extraction and high-throughput sequencing. In this experiment, the healthy taproot (primary root) is represented as HG. HX represents the healthy fibrous root, DG the diseased (root rot) primary root, and DX the root rot fibrous root of *C. chinensis*.

### Rhizosphere soil.

The rhizosphere soil of healthy and diseased *C. chinensis* plants was cultivated for 3 years and soil never planted with *C. chinensis* was used in this study. Coptis rhizosphere soil samples were collected (by shaking the root) during the peak prevalence of root rot disease (August each year). Each soil sample of the three treatment groups was collected at five points, and at least 10 Coptis plants were taken from each sampling point to be mixed as one sample. The plant residue in the soil was picked out with sterilized tweezers and transferred into sterilized bags, which were then divided into two parts. One part was stored at –80°C for DNA extraction and microbiome sequencing. The other part was dried in the shade at room temperature and used for the determination of soil nutrients after sieving. At the same time, the surrounding soil (in the same area) that has not been planted with Coptis was also collected, and the surface of the soil layer was carefully cleaned. Five-point sampling was carried out at a depth of5 to 15cm. The treatment of mixed samples is the same as that of rhizosphere soil sampling. The rhizosphere soils of healthy, root-rotted and unplanted *C. chinensis* soils were denoted as H. RMS3, D. RMS3, and N. RMS3, respectively.

### RNA sequencing and differential expression genes analysis.

Total RNA was extracted from the taproot and fibrous root of *C. chinensis* using the plant total RNA extraction kit (Sigma-Aldrich, STRN50-1KT) according to the manufacturer's protocol. Illumina's TruSeq Stranded mRNA LTSample Prep kit (Illumina, USA) was used to construct libraries of copitis. The 12 cDNA libraries of Copitis were sequenced on the Illumina sequencing platform (Illumina HiSeq X 10) to generate the 125 bp/150 bp paired-end reads. Transcriptome profiling and analysis were followed by Liu et al. (48). Raw reads (raw data) were cleaned in order to remove adapters, Ploy-N reads, and low-quality reads to obtain clean reads. Through HISAT2, all clean reads were mapped to the reference genome for subsequent analyses. An analysis of the FPKM values was calculated by using the log_2_ transformation and the read counts of each gene were obtained by htseq-count. The DEGseq R package was used to compute and analyze differentially expressed genes (DEGs) with a |log_2_ FC| > 1 and Q 0.005 threshold. Kyoto Encyclopedia of Genes and Genomes (KEGG) and Gene ontology (GO) enrichment analyses of DEGs were conducted with R based on the hypergeometric distribution during root rot pathogen infection in copitis.

### Determination of alkaloid content in *Coptis chinensis*.

The seven alkaloids determination of columbamine, epiberberin, coptisine, palmatine chloride, berberin, thaliotrine, and jatrorrhizine in Rhizoma Coptis were followed by Peng et al. (49) with slight modification. Chromatographic condition: the HPLC (Shimadezu DGU-20A) was performed on a Xtimate TM _C18 column_ (4.6 mm × 250 mm, 5 μm). The mobile phase was a mixture of acetonitrile and 30 mmol/L ammonium bicarbonate (which contains 0.7% ammonia water and 0.1% triethylamine). Linear gradient elution: the detection wavelength was 270 nm, the column temperature was 30°C, and the flow rate was 1.0 mL/min.

### Detection of rhizosphere microorganisms in healthy and diseased Coptis.

The total microbial DNA samples were extracted from 1 g soil samples (dry weight) using the Fast DNA Spin kit for Soil (MP Biomedicals, USA) following the manufacturer’s protocols. Each sample was a mixture of 30 DNA from every treated soil sample. In order to determine the final DNA concentration and purification, we used a NanoDrop 2000 UV-visible spectrophotometer (Thermo Scientific, USA), and for quality assurance was used a 1% agarose gel electrophoresis. Then, the DNA was diluted to 1 ng/μL and kept at –80°C for the fungal internal transcribed spacer (ITS) gene amplification. The V3-V4 regions of the 16S rDNA gene of the rRNA were amplified according to Tan’s et al. (50) method. The fungal ITS2 region was amplified with the ITS3_KYO2 and ITS4 primers in this study. PCRs were conducted following a previously described protocol (11). Then, the 2% agarose gels and Trelief DNA Gel Extraction kit (TSINGKE, TSP601-200, China) were used to extract and purify PCR amplicons according to the manufacturer’s instructions, respectively. The purified amplicons were pooled in equimolar amounts and sequenced on an Illumina platform using standard protocols (51). The potato dextrose agar medium was used to isolate root rot pathogens in *C. chinensis*.

### Data availability.

The raw data sequence and supporting sample-specific information discussed in this article have been deposited in the National Center for Biotechnology Information (NCBI) data set accession number (PRJNA916833). We confirm that all materials and experiments in this study were carried out in accordance with the relevant guidelines and regulations.
