# Supplementary material for: Multi-Omics Analysis Reveals the Resistance Mechanism and the Pathogens Causing Root Rot of Coptis chinensis
Source: Microbiol Spectr. 2023 Feb 21;11(2):e04803-22. doi: 10.1128/spectrum.04803-22 (PMC10101010; doi:10.1128/spectrum.04803-22)
Supplement: Supplemental file 1 — Figures S1 and S2. Download spectrum.04803-22-s0001.pdf, PDF file, 0.2 MB [file spectrum.04803-22-s0001.pdf]

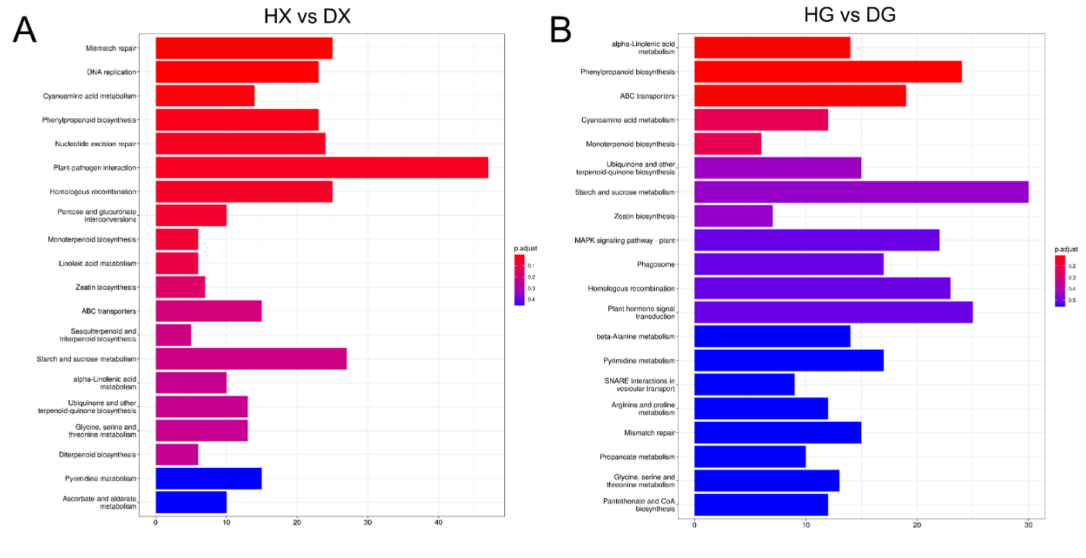

Figure S1. KEGG analysis of differentially expressed genes (DEGs) in healthy and root rot roots of *C. chinensis*. (A). HX vs DX comparable group; (B) HG vs DG comparable group. In the figure, HG and HX represent the primary root and fibrous root of healthy Coptidis, respectively. DG and DX represent the primary root and fibrous root of Coptidis, respectively.

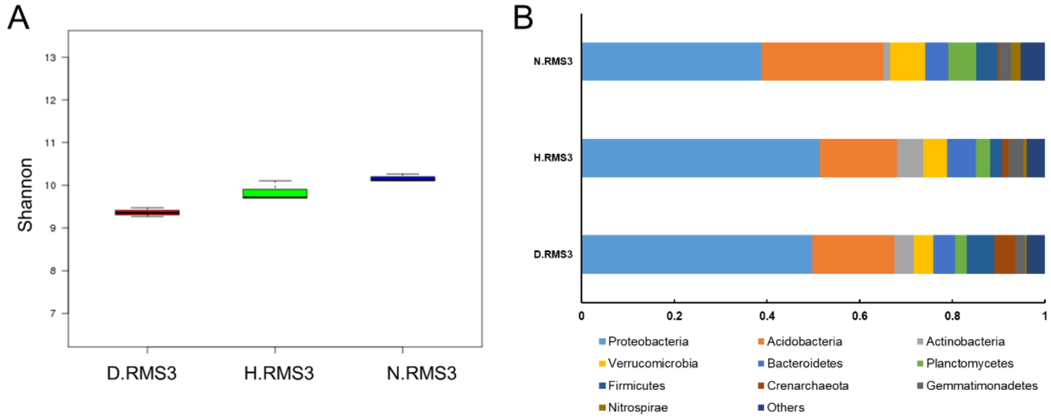

Figure S2. The bacterial α diversity index analysis in each rhizosphere soil of *Coptis coptidis* (A), and the relative abundance of soil microorganisms in each rhizosphere soil (B). N. RMS3 was not been planted *C. chinensis* soil. H. RMS3 and D. RMS3 were cultivation of health and root rot *C. chinensis* soil, respectively.
